# Glossopharyngeal and Hypoglossal Nerve Paralysis Secondary to Prevertebral Phlegmon

**DOI:** 10.1155/2020/3795035

**Published:** 2020-02-11

**Authors:** Ryunosuke Fukushi, Izaya Ogon, Yoshinori Terashima, Hiroyuki Takashima, Tsutomu Oshigiri, Noriyuki Iesato, Mitsunori Yoshimoto, Makoto Emori, Atsushi Teramoto, Toshihiko Yamashita

**Affiliations:** Department of Orthopedic Surgery, Sapporo Medical University School of Medicine, Sapporo, Japan

## Abstract

A 50-year-old man presented to the clinic with severe neck pain, fever, and difficulty breathing and was subsequently admitted to the local orthopedics department with possible retropharyngeal abscess and pyogenic spondylitis. Antibiotic therapy was initiated; however, due to poor oxygenation, he was referred and transferred to our department and admitted. Magnetic resonance imaging showed signal changes at the left C1/2 lateral atlantoaxial joint, posterior pharynx, longus colli muscle, carotid space, and medial deep cervical region, predominantly on the left side. In addition, despite lymph node enlargement from the posterior pharynx to the deep cervical region, there was no abscess formation. There were no signs of a space-occupying lesion or signal changes in the jugular foramen. One day postadmission, the patient's temperature had risen to 39.1°C and his SpO_2_ had fallen. His neck pain had also worsened, and emergency surgery was decided. Preoperatively, we suspected retropharyngeal abscess and pyogenic spondylitis. On day 13 postadmission, the patient exhibited dysphagia, deviated tongue protrusion, and the curtain sign. Glossopharyngeal and hypoglossal nerve paralysis were diagnosed. The patient's swallowing functions recovered and he was discharged on day 36. We experienced a case of glossopharyngeal and hypoglossal nerve paralysis secondary to pyogenic cervical facet joint arthritis.

## 1. Introduction

Jugular foramen syndrome is a rare systematic cranial neuropathy that presents characteristic clinical symptoms due to the paralysis of jugular foramen cranial nerves. In addition, a lesion in the carotid space, which surrounds the common carotid and internal carotid arteries, can cause cranial neuropathy. Although both jugular foramen syndrome and carotid space lesions have been reported rarely in patients treated in otolaryngology, no cases have been reported in the orthopedic field. Here, we report a case of cranial neuropathy caused by prevertebral phlegmon.

## 2. Case Report

A 50-year-old man with severe neck pain, fever, and difficulty breathing and no initial cause was later diagnosed with cervical spondylosis deformans and prescribed analgesics. Neck pain worsened after 5 days, and he was examined for suspected meningitis, which was ruled out. He was admitted to the local orthopedics department to diagnose a possible retropharyngeal abscess with pyogenic spondylitis. Antibiotic therapy was initiated; however, due to poor oxygenation, he was transferred to our department the same day and admitted to the intensive care unit (ICU). Upon hospitalization, his body temperature was 38.7°C and SpO_2_ was 92% (transnasal, 2 L). Physical findings included neck pain at rest and upon movement along with redness, swelling, feeling of warmth, and pressure around the entire neck. There were no signs of motor paralysis, sensory disturbances, abnormal deep tendon reflexes, or pathological reflexes. A blood test revealed leukocyte and C-reactive protein (CRP) levels of 20,900/*μ*L and 25.25 mg/dL, respectively.

**Figure 1:**
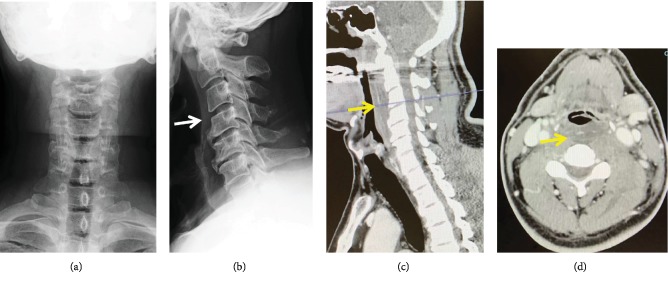
Initial radiographic and computed tomography (CT) assessment of the cervical spine. Posteroanterior (a) and lateral plane (b) radiograms. A lateral plane radiogram showed soft tissue swelling anterior to the vertebral bodies (white arrow). Sagittal (c) and axial (d) CT indicated soft tissue swelling in the posterior pharynx (yellow arrows), but there were no clear signs of abscess formation.

**Figure 2:**
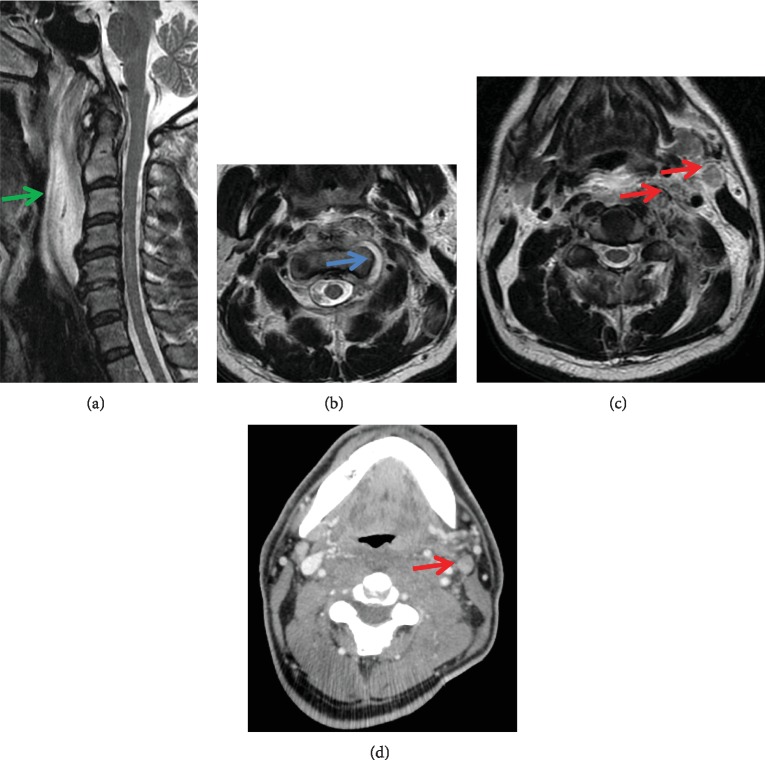
Initial magnetic resonance imaging (MRI) assessment of the cervical spine. Sagittal (a) and axial (b–d) MRI showed signal changes at the left C1/2 lateral atlantoaxial joint (blue arrow), posterior pharynx (green arrow), longus colli muscle, carotid space, and the medial deep cervical region, predominantly on the left side. Lymph node enlargement (red arrows) was observed from the posterior pharynx to the deep cervical region; there was no abscess formation.

**Figure 3:**
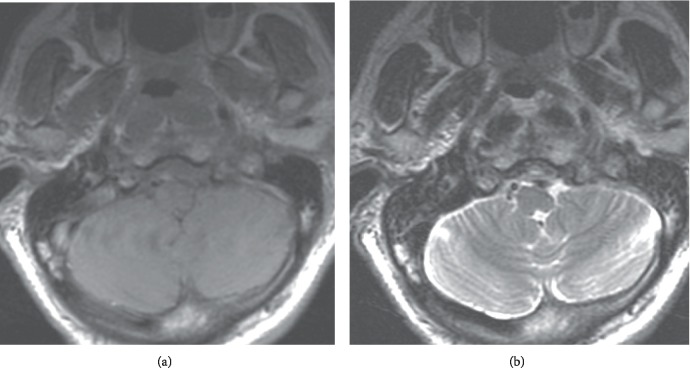
MRI assessment of the jugular foramen. Sagittal (a) and axial (b) MRI showed no signs of a space-occupying lesion or signal changes in the jugular foramen.

On X-ray examination at admission, soft tissue swelling was noted anterior to the vertebral bodies. Contrast-enhanced computed tomography revealed soft tissue swelling in the posterior pharynx without any clear signs of abscess formation (Figures 1(a)–1(d)). Magnetic resonance imaging (MRI) demonstrated signal changes at the left C1/2 lateral atlantoaxial joint, posterior pharynx, longus colli muscle, carotid space, and medial deep cervical region, predominantly on the left side. In addition, lymph node enlargement was observed from the posterior pharynx to the deep cervical region; however, no abscess formation was noted (Figures 2(a)–2(d)). In other image slices, there were no signs of space-occupying lesions or signal changes in the jugular foramen (Figures 3(a) and 3(b)).

Otorhinolaryngological examination revealed that the patient was able to converse and had no airway obstructions, stridor, or difficulty in swallowing. Examination using a fiberscope indicated swelling of the posterior oropharyngeal wall but revealed no abscess or other abnormal findings. The blood culture was positive for *Staphylococcus aureus*, but the results of other rapid tests were negative.

One day postadmission, the patient's temperature increased to 39.1°C and his SpO_2_ decreased. Moreover, his physical symptoms deteriorated with worsening neck pain, increased blood CRP levels, and impaired respiratory status. Therefore, emergency surgery was performed. Imaging findings indicated that the likelihood of abscess formation was low; however, urgent, experimental surgery was performed to make a definitive diagnosis. Since the patient exhibited fever and a sharp rise in the inflammatory response, we also aimed to identify the bacteria responsible for the inflammation. An anterior cervical approach was selected for incision, drainage, and irrigation.

Intraoperative findings revealed no abscess formation in the posterior pharynx or vertebral bodies, or anterior to the intervertebral discs. Irrigation was performed with a large amount of physiological saline, and a drain was placed. The patient was then admitted to the ICU without being extubated and fitted with an Aspen® Cervical Collar.

Postoperatively, white blood cell (WBC) count and CRP levels exhibited a decreasing trend. On day 4 of hospitalization, he was extubated and released from the ICU, and the drain was removed on day 9. A catheter tip culture was positive for *S. epidermidis*; this result differed from that of the blood culture. This false positive was thought to be from contamination. The patient's previous physician had administered IPM/CS; however, based on the blood culture results of the previous physician, the patient's treatment regimen was changed to ABPC/CVA after being transferred to our hospital. Continued administration of antibiotics gradually decreased WBC count and CRP level. On day 13 of hospitalization, the patient had dysphagia, deviated tongue protrusion, and the curtain sign. Based on these findings, glossopharyngeal and hypoglossal nerve paralysis were diagnosed. Despite the presence of glossopharyngeal and hypoglossal nerve paralysis, WBC and CRP levels were negative and the patient was switched to antibiotic administration via a gastric tube.

The patient's swallowing functions recovered over the next month in the hospital, and he was discharged on day 36. An MRI was performed on postoperative day 68 while the patient was still hospitalized; signal change was observed in the facet joint, suggesting that swelling had subsided. At the 3-month follow-up, there was no recurrence, and radiographic images showed no abnormal findings.

## 3. Discussion

Until now, three studies have reported the incidence of glossopharyngeal and hypoglossal nerve paralysis caused by infection. Ohara et al. reported a case of glossopharyngeal, vagal, and hypoglossal nerve paralysis that recovered with antibiotics in a 1-year-old boy [1]. They reported that the paralysis was caused by pressure on the carotid space. Miyamoto et al. reported a 62-year-old woman with glossopharyngeal nerve paralysis, as well as vagal and accessory nerve paralysis [2]. The causative pathogen in the aforementioned case was *Mycobacterium tuberculosis*; although the patient was treated with antitubercular agents, some vocal cord paresis remained. Shiratsuchi et al. reported a 71-year-old man with glossopharyngeal and hypoglossal nerve paralysis [3]. In this case, the causative agent was a fungus and the patient's condition improved with the use of an antifungal agent. Miyamoto et al. and Shiratsuchi et al. reported that paralysis was caused by jugular foramen syndrome.

The jugular foramen is located on the inferior surface of the temple and acts as a pathway for the internal jugular vein and glossopharyngeal, vagal, and accessory nerves. If the jugular foramen is damaged by infection, trauma, cerebrovascular accident, and polyangiitis or is occupied by tumor, dysfunction of the glossopharyngeal, vagal, or accessory nerves can result [3, 4]. The carotid space is present in the region of the common carotid artery and internal carotid artery. Moreover, its height extends from the jugular foramen to the aortic arch, cervicofacial area, and superior mediastinum. The carotid space is anteriorly, laterally, medially, and posteriorly surrounded by the pharyngeal space, parotid gland, retropharyngeal space, and anterior vertebrae, respectively [5, 6]. In addition, glossopharyngeal and hypoglossal nerve dysfunction can result from pressure arising from lymph node enlargement due to infection, tumors, or other pathologies [5].

Jugular foramen syndrome does not generally involve damage to the hypoglossal nerve [7–11]; however, pressure exerted on the carotid space may lead to hypoglossal nerve dysfunction. In the present case, the patient had hypoglossal nerve paralysis, which made us believe that the pathogenesis was due to pressure on the carotid space.

The lateral retropharyngeal lymph nodes (LRPLNs) are present on the medial side of the carotid space, which contains the glossopharyngeal and hypoglossal nerves [5]. Ohara et al. demonstrated that infection led to LRPLN enlargement, which resulted in pressure on the carotid space from the medial to lateral side, causing glossopharyngeal and hypoglossal nerve paralysis. The lack of symptoms associated with the accessory, vagal, and sympathetic nerves, which also pass through the carotid space, may be explained by the location of these tissues' pathways. They are on the dorsal side of the space and are thus less likely to be affected by pressure from an abscess [1]. We believe that the pathogenesis in the present case was through the same mechanism.

Further enlargement of LRPLN may have caused symptoms involving the accessory, vagal, and sympathetic nerves. To our knowledge, this is the first study to report a case of cranial neuropathy caused by prevertebral phlegmon in orthopedics.

The current case report had several limitations. We did not perform direct suctioning from the facet joint, or gadolinium in MRI. One significant limitation of this study is that we were unable to confirm the actual location of the compression. However, we surmised the pathological diagnosis of this case based on several findings including the absence of abscesses and the presence of lymphadenopathy near the carotid space on images, and by citing previous literature.

## 4. Conclusions

We report a case of glossopharyngeal and hypoglossal nerve paralysis that occurred secondary to prevertebral phlegmon. Although the presence of an abscess was not confirmed in the images or intraoperative findings, the patient's symptoms improved with antibiotic administration. We believe lymph node enlargement due to inflammation resulted in pressure on the carotid space, which affected the functioning of the glossopharyngeal and hypoglossal nerves passing through this space.
